# SABRE hyperpolarization enables high-sensitivity ^1^H and ^13^C benchtop NMR spectroscopy†
†Electronic supplementary information (ESI) available. See DOI: 10.1039/c8an00596f


**DOI:** 10.1039/c8an00596f

**Published:** 2018-06-19

**Authors:** Peter M. Richardson, Andrew J. Parrott, Olga Semenova, Alison Nordon, Simon B. Duckett, Meghan E. Halse

**Affiliations:** a Centre for Hyperpolarisation in Magnetic Resonance , Department of Chemistry , University of York , UK . Email: meghan.halse@york.ac.uk ; Email: simon.duckett@york.ac.uk; b WestCHEM , Department of Pure and Applied Chemistry and CPACT , University of Strathclyde , Glasgow , UK

## Abstract

Benchtop NMR spectrometers operating with low magnetic fields of 1–2 T at sub-ppm resolution show great promise as analytical platforms that can be used outside the traditional laboratory environment for industrial process monitoring. One current limitation that reduces the uptake of benchtop NMR is associated with the detection fields’ reduced sensitivity. Here we demonstrate how *para*-hydrogen (*p*-H_2_) based signal amplification by reversible exchange (SABRE), a simple to achieve hyperpolarization technique, enhances agent detectability within the environment of a benchtop (1 T) NMR spectrometer so that informative ^1^H and ^13^C NMR spectra can be readily recorded for low-concentration analytes. SABRE-derived ^1^H NMR signal enhancements of up to 17 000-fold, corresponding to ^1^H polarization levels of *P* = 5.9%, were achieved for 26 mM pyridine in *d*_4_-methanol in a matter of seconds. Comparable enhancement levels can be achieved in both deuterated and protio solvents but now the SABRE-enhanced analyte signals dominate due to the comparatively weak thermally-polarized solvent response. The SABRE approach also enables the acquisition of ^13^C NMR spectra of analytes at natural isotopic abundance in a single scan as evidenced by hyperpolarized ^13^C NMR spectra of tens of millimolar concentrations of 4-methylpyridine. Now the associated signal enhancement factors are up to 45 500 fold (*P* = 4.0%) and achieved in just 15 s. Integration of an automated SABRE polarization system with the benchtop NMR spectrometer framework produces renewable and reproducible NMR signal enhancements that can be exploited for the collection of multi-dimensional NMR spectra, exemplified here by a SABRE-enhanced 2D COSY NMR spectrum.

## Abstract 2

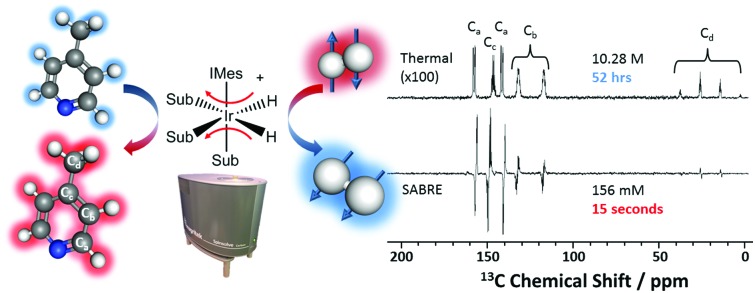
Benchtop NMR spectrometers operating with magnetic fields of 1–2 T at sub-ppm resolution coupled with SABRE hyperpolarization show great promise as analytical platforms that can be used outside the traditional laboratory environment.

## Introduction

Nuclear magnetic resonance (NMR) spectroscopy is a vitally important analytic science tool that probes the chemical and physical properties of matter and whose timescales makes it well suited for reaction monitoring. When compared to other analytical techniques, such as optical spectroscopy and mass spectrometry, NMR is however inherently insensitive.^1^ The sensitivity associated with an NMR measurement is proportional to the population difference across the nuclear spin states it probes (the so-called polarization), which in turn is dependent on the magnetic field strength of the spectrometer, *B*_0_. ^1^H nuclei have a field-dependent polarization level of just 3.5 ppm T^–1^ at room temperature which drives the need to involve large magents.^1^ In fact to maximize sensitivity, modern NMR uses highly expensive superconducting magnets with fields ranging from 7 to 23 T. These high-field NMR spectrometers are not ideal for industrial process monitoring, as they require costly cryogens and expert technical support, have a large footprint, are expensive, and non-portable.^2^,^3^ One potential solution is to use low-field NMR spectrometers, based on either electromagnets or permanent magnet arrays.^4^–^6^ While such compact NMR spectrometers are considerably less expensive, more portable, and more robust than superconducting magnets, requiring minimal maintenance, they clearly have low sensitivity. Notwithstanding this, low-field NMR devices have well established applications in the food^7^ and oil and gas^8^ industries for the measurement of relaxation rates and molecular diffusion coefficients where chemical shift information is not required. More recently, benchtop NMR spectrometers based on permanent magnet arrays with magnetic fields of 1–2 T with sub-ppm homogeneity have become available^9^ and the range of analytical applications is growing.^5^,^10^–^12^

One of the main drawbacks of benchtop NMR is the low sensitivity. In this work we overcome this limitation through the use of hyperpolarization.^13^ Hyperpolarization is a general term that refers to the generation of a nuclear spin alignment that is significantly larger than that dictated by the Boltzmann distribution at thermal equilibrium.^14^,^15^ There are various approaches to hyperpolarization, each with different strengths and weaknesses. Some of the most popular methods include: dynamic nuclear polarization (DNP),^16^,^17^ spin-exchange optical pumping (SEOP),^18^ and *para*-hydrogen (*p*-H_2_) induced polarization (PHIP).^19^–^26^ In the context of low-field NMR spectroscopy, the ideal hyperpolarization method needs to be low-cost, fast, and relatively simple to implement so that the overall cost and portability advantages of compact NMR are not compromised.^13^ In this work, we focus on the PHIP approach where the source of hyperpolarization, *p*-H_2_, is relatively easy and cheap to access.^27^–^30^

Molecular hydrogen, H_2_, has two nuclear spin isomers: the triplet state, *ortho*-hydrogen, and the singlet state, *p*-H_2_. *p*-H_2_ has no net angular momentum and so is NMR silent. In order to exploit the spin order that is stored within *p*-H_2_ the symmetry of the *p*-H_2_ molecule must be broken, typically through a chemical reaction. In the original PHIP experiments of Bowers and Weitekamp (PASADENA and ALTADENA) visible hyperpolarization is generated through hydrogenation of an unsaturated precursor.^27^,^31^,^32^ This leads to dramatic NMR signal enhancements on the product molecule. However, the need to generate hyperpolarized molecules *via* hydrogenation significantly limits the scope of this approach to a relatively narrow range of chemicals with a suitable chemical precursor. In this work we focus on the signal amplification by reversible exchange (SABRE) method, which uses *p*-H_2_ as a source of hyperpolarization but does not require hydrogenation of the target molecule, providing renewable hyperpolarization over a timescale of tens of seconds with the addition of fresh *p*-H_2_.^19^,^20^

**Fig. 1:**
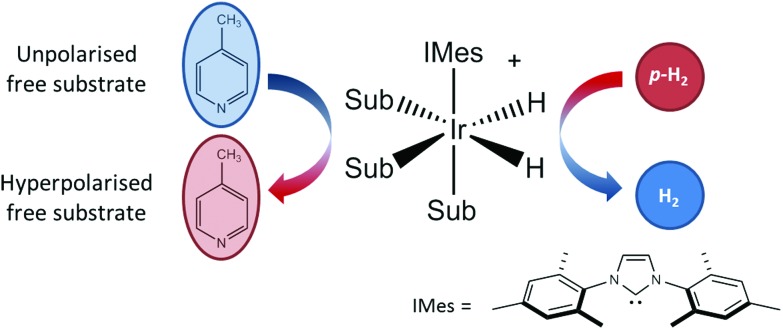
An active SABRE catalyst reversibly binds both *p*-H_2_ and a substrate to allow polarization transfer from *p*-H_2_ to the substrate in a polarization transfer field (PTF) of a few tens of gauss.

As illustrated in Fig. 1, SABRE is essentially a catalytic polarization transfer process. It works *via* the transient binding of the *p*-H_2_ and target substrate(s) to a transition-metal complex to form a *J*-coupling network that permits the transfer of spin order from the *p*-H_2_ to the nuclei on the substrate of interest.^19^ This transfer is achieved by carrying out the exchange reaction in a small magnetic field referred to as the polarization transfer field (PTF). For optimal polarization transfer in SABRE, the dominant *J*-coupling interaction in the active complex needs to be comparable to the chemical shift difference between the ^1^H derived from *p*-H_2_ and the target substrate nuclei.^28^,^33^ This corresponds to a PTF of tens of gauss for transfer to ^1^H,^34^ with much lower PTF values required for direct transfer to heteronuclei such as ^15^N and ^13^C.^22^

The conditions for efficient SABRE hyperpolarization are that the *p*-H_2_ and substrate reversibly bind to the active catalyst and the two *p*-H_2_-derived hydride nuclei couple differently to the bound substrate nuclei.^28^ The rates of exchange of the substrate and *p*-H_2_, along with the NMR relaxation times (*T*_1_) of the system and the propagating spin–spin couplings, dictate the maximum level of polarization that can be built up.^35^ It has been found that N-heterocycles such as pyridine are efficient SABRE targets when used with catalysts such as [IrCl(COD)(IMes)] (where COD = 1,5 cyclooctadiene and IMes = 1,3-bis (2,4,6-trimethyl-phenyl)-imidazolium).^36^ Recent work has shown that deuteration of the catalyst can result in ^1^H hyperpolarization levels of up to 50%.^37^ In addition, the scope of molecules amenable to hyperpolarization using the SABRE approach has recently been dramatically increased to include molecules with any functional group containing an exchangeable proton through the introduction of the SABRE-Relay mechanism.^38^ We focus here on the traditional SABRE method, but these developments will transfer directly to the novel SABRE-Relay approach to achieve a truly versatile platform to transform low-field analysis.

SABRE itself has already been shown to hyperpolarize a wide range of nuclei including ^19^F,^39^^31^P,^40^,^41^ ^119^Sn,^42^^13^C^19^,^43^–^45^ and ^15^N.^22^,^33^,^46^,^47^ For benchtop NMR, natural abundance ^13^C{^1^H} spectra are of particular interest because, unlike ^1^H spectra acquired at 43 MHz, which suffer from significant peak overlap and second-order coupling patterns due to limited chemical shift dispersion, ^13^C{^1^H} NMR spectra at 43 MHz and 400 MHz are often virtually identical in terms of observed spin-dilute peak patterns. However, due to the lower gyromagnetic ratio and very low natural abundance (1.109%) of ^13^C, the signal strength is very weak and so concentrated samples or isotopic labelling coupled with many transients (*i.e.* long experiment times) are often required.

^13^C SABRE hyperpolarization can also be achieved through direct transfer of polarization from the *p*-H_2_-derived hydrides of the active SABRE complex (Fig. 1) to the ^13^C nuclei of the substrate.^33^ In this case, polarization transfer fields of around 0.25 μT (or 2.5 mG), achieved through the use of mu-metal shields to exclude the Earth's magnetic field, are required to fulfil the necessary resonance condition.^22^,^33^ Due to the coupling between the ^1^H and ^13^C nuclei, it is also possible to hyperpolarize two-spin-order states involving ^1^H and ^13^C on the substrate. This type of hyperpolarization can be generated in PTFs similar to the ^1^H SABRE experiments (*i.e.* tens of G).^48^

^1^H SABRE hyperpolarization has been applied to compact NMR systems with detection field strengths ranging from tens of mT^49^–^53^ down to zero to ultra-low field.^54^–^58^ SABRE-hyperpolarized ^13^C NMR has also been demonstrated in a field of 5.75 mT, where very high NMR signal enhancements were observed (*e.g.* 30 000 000 in the ^13^C case); however no chemical shift information is available in this field regime.^59^ SABRE hyperpolarized ^1^H NMR spectra have also recently been shown for benchtop NMR spectrometers with fields of 1–2 T with sub-ppm chemical shift resolution using single-shot acquisition.^13^,^60^,^61^

In this work we explore the combination of SABRE hyperpolarization with a 1 T (43 MHz) benchtop NMR spectrometer using both manual and automated flow-based approaches. We compare the SABRE hyperpolarization levels that are achieved with high (9.4 T) and low-field (1 T) detection, explore the effect of protio solvents on the observed enhancements at low field, and demonstrate single-scan SABRE-hyperpolarized ^1^H and ^13^C benchtop NMR spectra. We explore the reproducibility of the renewable SABRE hyperpolarization using an automated flow and illustrate how this system can be used to achieve SABRE hyperpolarized 2D NMR spectroscopy.

## Experimental

Two different methods for generating SABRE hyperpolarization were explored. First is a manual approach where SABRE is carried out in an NMR tube fitted with a Young's valve. The *p*-H_2_ gas is added to the head-space of the NMR tube at a pressure of 4 bar. The tube is subsequently shaken (for 4 seconds for ^1^H and 10 seconds for ^13^C measurements) in a polarization transfer field (PTF) of either 63 G (^1^H) or the ambient Earth's magnetic field of ∼50 μT (^13^C). The 63 G PTF is generated by a Halbach array as described previously.^61^ The NMR tube is then rapidly transferred into either the benchtop or 400 MHz NMR spectrometer for signal detection. Typical transfer times are approximately 3 s and 5 s for the benchtop and 400 MHz spectrometers, respectively. The *p*-H_2_ is generated using an apparatus operating at a conversion temperature of 28 K (∼98% enrichment). Fresh *p*-H_2_ is added to the NMR tube between SABRE experiments by evacuating the head-space before refilling with 4 bar *p*-H_2_.

In the second approach, *p*-H_2_ is bubbled through the sample at 4 bar in a PTF between 0 and 140 G produced by an electromagnet. Following a release of the *p*-H_2_ pressure, the solution is flowed into the benchtop NMR spectrometer under a pressure of nitrogen gas for signal detection. The automated flow system has been described previously for use with high-field NMR spectrometers^48^,^62^ and has been adapted here for use with a 1 T (43 MHz) NMR spectrometer (Spinsolve Carbon, Magritek). The full system consists of a hydrogen electrolysis cell (Peak Scientific), a *p*-H_2_ generator operating at 38 K to provide an estimated 92% *p*-H_2_ enrichment (Bruker), a mixing chamber within an electromagnet (a solenoid), and a glass cell inside the benchtop NMR spectrometer. The mixing chamber and flow cell are connected using fluorinated ethylene propylene (FEP) tubing. The glass flow cell was custom designed and built in house using a 30 mm section of a high quality NMR tube incorporated into the middle of a glass capillary (outer diameter of 4.3 mm and internal diameter of 2 mm). The lengths of the capillary were such that the NMR tube section was located in the detection region of the NMR instrument. The sample was transferred between the mixing chamber and the flow cell using a pneumatic control unit fed with nitrogen gas (5 bar). The pneumatic control unit (Bruker) allows for the return of the sample to the external mixing chamber after measurement. The flow cell has an exhaust to regulate the pressure during the transfer steps.

All 1D ^1^H NMR spectra were acquired following a single broadband 90° RF pulse. The 1D ^13^C NMR spectra were obtained either (a) following a single broadband 90° RF pulse without decoupling or (b) following an echo-based refocusing sequence and interleaved broadband ^1^H decoupling, see ESI† for details.^27^ The 2D COSY was acquired using a standard gradient selective COSY sequence that was modified to allow for repolarization by SABRE between each step of the experiment. For each mixing time, a single transient was acquired following: bubbling of *p*-H_2_ through the solution within the mixing chamber for 15 seconds, a 3 s delay for the release of the *p*-H_2_ pressure, 0.9 s for transfer of the sample to the spectrometer and a 0.1 s settling time inside the spectrometer. Following acquisition, the sample was returned to the mixing chamber. A delay of 10 s was included before each repolarization step to allow for full recovery of *p*-H_2_ pressure within the generator.

All samples used 5.2 mM [IrCl(COD)(IMes)] pre-catalyst loading with varying concentrations of substrate. The pre-catalyst and substrate were added to either 0.6 mL (for the manual approach) or 3 mL (for the automated approach) of methanol and mixed until fully dissolved. Both methanol-*d*_4_ (CD_3_OD) and protonated methanol (CH_3_OH) were used (Sigma Aldrich). In all cases, the active catalyst form [Ir(H)_2_(IMes)(sub)_3_]Cl (Fig. 1), is generated after reaction of [IrCl(COD)(IMes)] with H_2_ and the substrate.^63^,^64^ In the case of the manual shaking method the activation procedure was to add H_2_ to the degassed sample and shake vigorously; the gas was then evacuated and replaced with fresh H_2_ gas. This was repeated 6 times over 10 minutes before being left for an additional 5 minutes to allow for complete activation. In the case of the flow system a comparable activation procedure was used, however, in this case the sample was injected into to the mixing chamber and H_2_ bubbled through the solution for 15 s, again repeated 6 times over a period of 10 minutes.

## Results and discussion

### ^1^H SABRE at 1 T and 9.4 T

SABRE is attractive for low-field NMR applications because the level of hyperpolarization is independent of the strength of the NMR detection field. To illustrate this effect, we compare SABRE hyperpolarization experiments using a conventional 9.4 T (400 MHz) NMR spectrometer and a 1 T (43 MHz) permanent magnet system for detection. We use pyridine as the analyte because it has been shown to yield large SABRE enhancement factors, and this substrate-catalyst system is well understood.^19^,^22^,^33^,^47^,^65^–^67^ The activated catalyst has the form [Ir(IMes)(H)_2_(py)_3_]Cl (where py = pyridine) shown in Fig. 1. Transfer of polarization from *p*-H_2_ proceeds spontaneously into the substrate molecules bound *trans* to the *p*-H_2_ hydrides.^36^

**Fig. 2:**
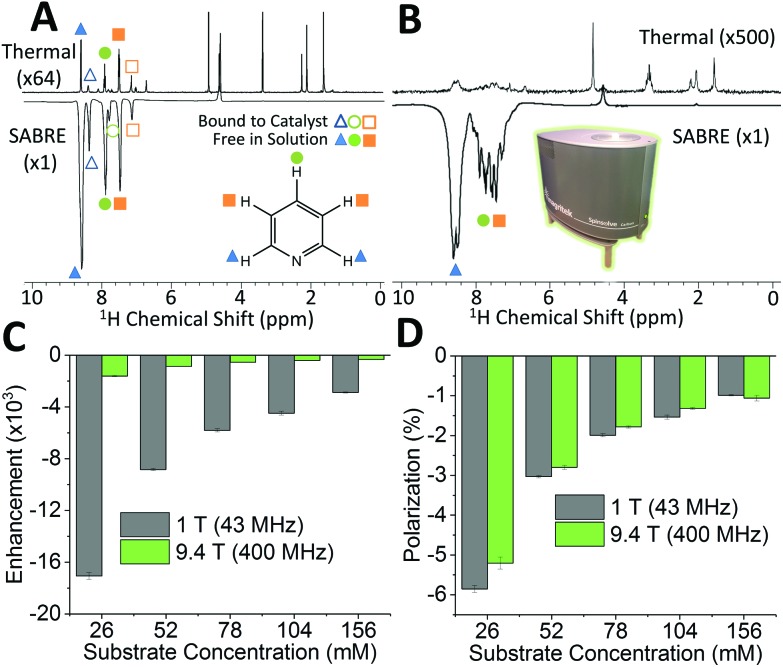
Comparison of thermally polarized (top) and SABRE hyperpolarized (bottom) ^1^H NMR spectra of 52 mM pyridine with 5.2 mM catalyst in methanol-*d*_4_ for NMR detection at (A) 9.4 T and (B) 1 T. (C) SABRE enhancement factor, *ε*, and (D) polarization level, *P*, for the *ortho* resonance (blue triangle) as a function of substrate concentration with NMR detection at 1 T (gray) and 9.4 T (green). Error bars represent the standard deviation across 5 measurements.

Fig. 2A shows a comparison between 400 MHz ^1^H NMR spectra acquired without (top) and with (bottom) SABRE hyperpolarization for a sample containing 52 mM pyridine with 5.2 mM of catalyst in methanol-*d*_4_. As expected, the hyperpolarized spectrum contains six enhanced resonances, corresponding to the three distinct ^1^H resonances of pyridine in free solution (solid shapes), and of *trans* pyridine bound to the catalyst (hollow shapes). Fig. 2B presents the same comparison for detection using a 43 MHz benchtop NMR spectrometer. It can be readily observed that the signal to noise has vastly increased in the hyperpolarized spectrum (bottom) relative to the thermal NMR case (top). However, unlike for high-field detection, only the ^1^H signal for the *ortho* protons of pyridine can be fully resolved, due to the reduced chemical shift dispersion at 1 T. The observed SABRE efficiency in the two detection fields can be evaluated by calculating the SABRE enhancement factor (*ε*), which is the ratio of the integral of the hyperpolarized spectrum, to the integral of the thermal NMR spectrum (see ESI†) as shown in Fig. 2C.

The enhancement factors observed at low field (1 T) are much higher than those at high field (9.4 T). This is to be expected because of the lower Boltzmann polarization at 1 T. To directly compare efficiency, we report the polarization level, *P*, obtained by scaling the Boltzmann polarization at thermal equilibrium in the detection field by the observed enhancement factor, *ε* (see ESI†). Fig. 2D confirms that comparable levels of polarization are observed for both detection fields. The maximum enhancement values observed for the *ortho* proton resonances were 1610 fold (5.2%) and 17 100 fold (5.9%) for the 9.4 T and 1 T detection fields, respectively. The higher polarization found for the benchtop measurement is attributed to the reduction in sample transfer time in the benchtop case, which reduces the loss of hyperpolarization due to NMR relaxation during transfer.

**Fig. 3:**
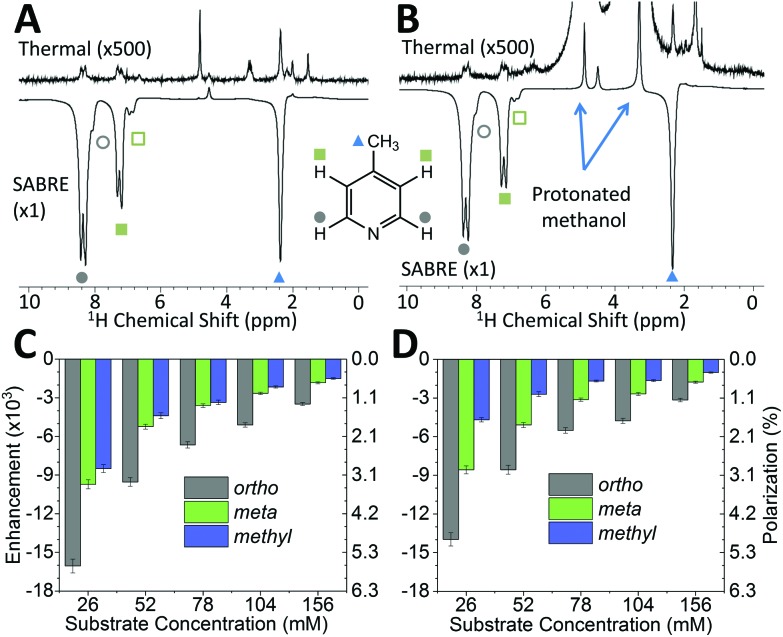
Comparison of benchtop (1 T) ^1^H NMR spectra acquired without (top) and with (bottom) SABRE hyperpolarization for 52 mM 4-methylpyridine and 5.2 mM catalyst in (A) methanol-*d*_4_ and (B) protonated methanol. SABRE enhancement factors and polarization levels as a function of substrate concentration in (C) methanol-*d*_4_ and (D) protonated methanol. The pre-catalyst concentration (5.2 mM) was kept constant in all cases. Enhancement factors and polarization levels are reported for the three distinct ^1^H resonances of the substrate: *ortho* (gray), *meta* (green) and *methyl* (blue). Error bars represent the standard deviation across 5 measurements.

While pyridine is an attractive test substrate for SABRE experiments due to its high levels of polarization, the reduced chemical shift dispersion of the benchtop NMR spectrometer gives rise to complicated ^1^H NMR spectra, with significant peak overlap in the aromatic region (Fig. 2B). This issue can be avoided through the use of *para*-substituted pyridine derivatives, such as 4-methylpyridine, as illustrated by the ^1^H NMR spectra at 1 T in Fig. 3A. As with pyridine, large SABRE signal enhancements are obtained, with the additional benefit that all three SABRE hyperpolarized ^1^H resonances are resolved at 1 T.

Fig. 3B presents thermally polarized and hyperpolarized ^1^H NMR spectra of 4-methylpyridine in protonated methanol. Remarkably, hyperpolarized analyte characterization is again possible. This contrasts with high-field NMR, where protonated solvents lead to dynamic range problems and radiation damping^68^ that results in the need for solvent suppression techniques.^69^–^71^ In the lower magnetic field the hyperpolarized analyte actually yields a larger response than that of the solvent. As illustrated by the enhancement factors presented in Fig. 3C (deuterated solvent) and Fig. 3D (protonated solvent), the efficiency of the SABRE hyperpolarization is comparable in both solvents, with the best enhancements in the two solvents being 16 000 fold (5.6%) and 14 000 fold (4.9%), respectively. The small systematic reduction in enhancement in the protonated solvent case can be attributed to NMR relaxation, which is typically faster when using protio solvents. The ability to use protonated solvents for these measurements is a significant benefit for industrial applications as it removes the need for costly sample preparation steps prior to analysis.

### Natural abundance ^13^C SABRE at 1 T

**Fig. 4:**
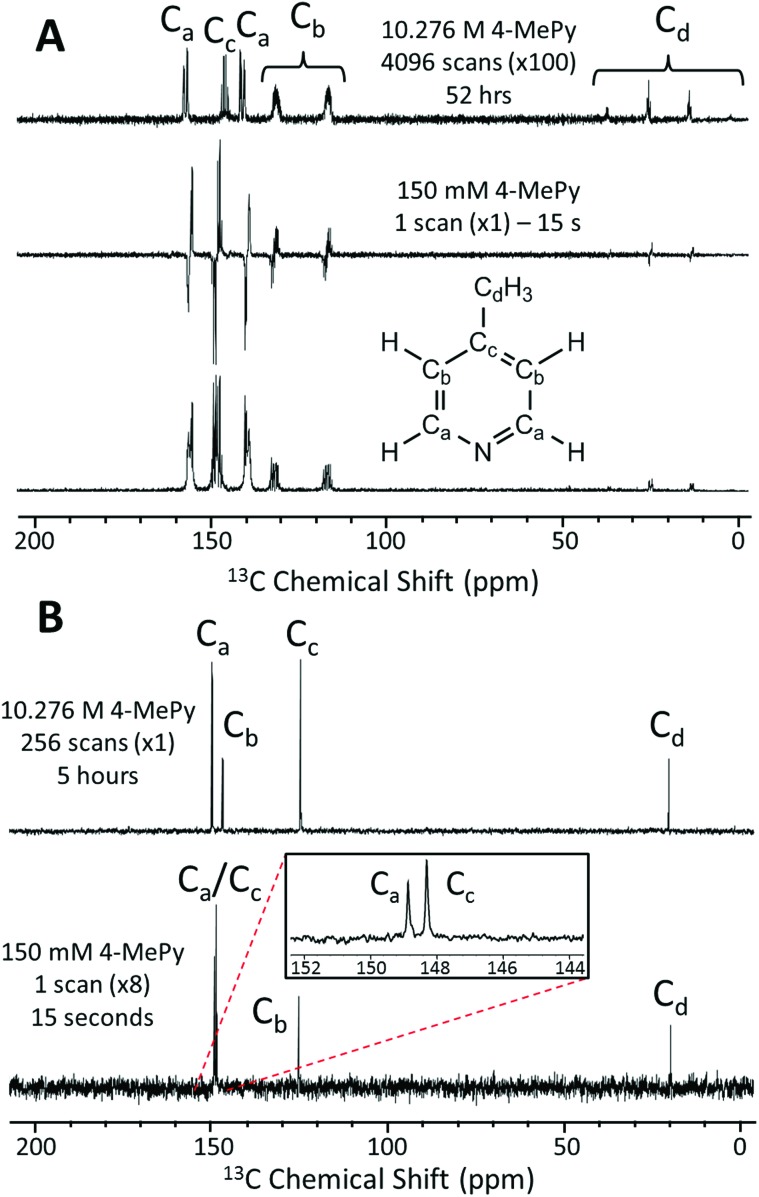
(A) Natural abundance ^13^C NMR spectra following a single 90° pulse for (top) 10.28 M (neat) 4-methylpyridine (4096 scans in 52 hours, scaled by ×100), (middle, bottom) 156 mM 4-methylpyridine with 5.2 mM catalyst in methanol-*d*_4_ (1 scan in 15 s with SABRE hyperpolarization), presented in real (middle) and magnitude (bottom) modes. (B) ^13^C{^1^H} NMR spectra acquired at 1 T of (top) 10.28 M (neat) 4-methylpyridine (256 scans in 5 hours) and (bottom) 156 mM 4-methylpyridine with 5.2 mM catalyst in methanol-*d*_4_ (1 scan in 15 s with SABRE hyperpolarization and refocusing prior to signal acquisition, scaled by x8).

In Fig. 4A we present a SABRE-hyperpolarized ^13^C NMR spectrum of 156 mM 4-methylpyridine at natural abundance. This spectrum was acquired in a total experiment time of only 15 seconds by manually shaking the sample for 10 s in the Earth's magnetic field (PTF ≈ 50 μT) prior to manually transferring it to the benchtop NMR spectrometer for signal detection. Here the Earth's magnetic field was observed to yield empirically larger enhancements when compared to the 63 G shaker and was thus chosen to exemplify the ^13^C hyperpolarization of this molecule. It is not possible to acquire a thermally-polarized NMR spectrum of a similarly dilute solution of the analyte at natural abundance. Therefore, a thermally polarized ^13^C NMR spectrum of neat 4-methylpyridine (10.28 M), acquired as the sum of 4096 transients in 52 hours, is provided for comparison (Fig. 4A, top). The hyperpolarized NMR spectrum is presented in both real (Fig. 4A, middle) and magnitude modes (Fig. 4A, bottom). In the real NMR spectrum, the ^13^C peaks are anti-phase relative to the two bond ^13^C–^1^H coupling (8–12 Hz). This suggests that the ^13^C signal arises from the hyperpolarization of a coupled ^1^H–^13^C two-spin-order term.

Using an appropriately scaled thermally polarized spectrum as a reference, the ^13^C enhancement factor is estimated to be *ε* = 13 700 (*P* = 1.2%) for the carbon in the *para* position (C_c_). Larger signal enhancement factors are observed at lower analyte concentrations, where the effective catalyst loading is higher and so SABRE is more efficient. A maximum enhancement factor of 45 500 fold (*P* = 4.01%) was observed for 26 mM of substrate with 5.2 mM of catalyst. See ESI† for full calculation and all enhancement values.

One of the major benefits of natural abundance ^13^C NMR is the relative simplicity of ^13^C{^1^H} spectra even in the low-field (1 T) regime. Fig. 4B shows a comparison of natural abundance ^13^C{^1^H} NMR spectra of neat 4-methylpyridine (top, thermally polarized, 256 scans in 5 h) and 156 mM 4-methylpyridine (bottom, SABRE hyperpolarized, 1 scan in 15 s). Prior to ^1^H decoupling, the anti-phase ^13^C signals were first refocused using an echo sequence (see ESI† for more details). The inset in Fig. 4B highlights the separation of the *ortho* and *para*^13^C resonances in the SABRE spectrum, despite a difference in chemical shift of <1 ppm. Note these peaks appear at different chemical shift values relative to the ^13^C{^1^H} spectrum of neat 4-methylpyridine due to solvent effects (see ESI† for more details). An interesting feature of the hyperpolarized ^13^C spectra is that the greatest SABRE enhancement is observed for the quaternary carbon (Fig. 4B, C_c_, *ε* = 17 000 and *P* = 1.5%); this can be attributed to its longer magnetic state lifetime, as there is no directly bound proton to drive relaxation. This is in contrast to standard ^13^C NMR, where these same factors make quaternary carbons the most difficult to detect.

### Automated SABRE experiments

**Fig. 5:**
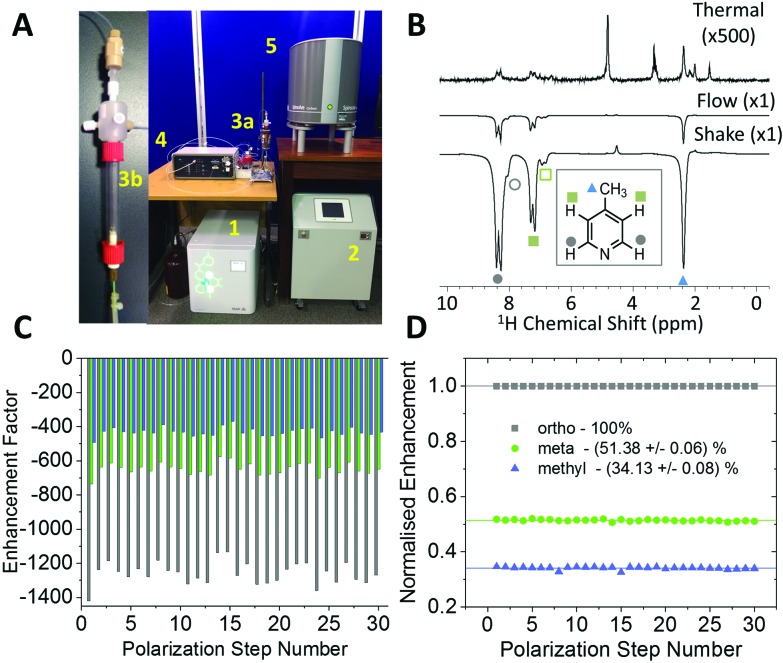
(A) Automated SABRE flow system that includes: (1) electrolysis cell, (2) Bruker *p*-H_2_ conversion unit, (3a) PTF solenoid coil, (3b) mixing chamber, (4) Bruker polarizer control unit, and (5) 43 MHz (1 T) Magritek Spinsolve Carbon NMR spectrometer. (B) ^1^H NMR spectra of 52 mM 4-methylpyridine with 5.2 mM catalyst in methanol-*d*_4_ acquired with (top) thermal polarization, (middle) SABRE hyperpolarization using the automated flow system, (bottom) SABRE hyperpolarization using manual shaking. Both SABRE spectra were acquired with a PTF ∼63 G. (C) Variability between repeated SABRE hyperpolarization experiments where the sample is repolarized between each acquisition. (D) Data from (A) normalized to the enhancement factor of the *ortho* resonance.

The experiments shown up to this point have been achieved using the manual shaking method. While this approach has been shown to provide a route to efficient and reproducible SABRE hyperpolarization, it cannot easily be extended to multi-dimensional experiments or signal averaging. For this an automated method is required. The automated system for SABRE hyperpolarization is pictured in Fig. 5A. Fig. 5B presents a comparison of 43 MHz ^1^H NMR spectra acquired with thermal polarization (top), the automated SABRE approach (middle) and SABRE with manual shaking (bottom). The observed SABRE enhancements are consistently lower for the automated flow system when compared to the manual shaking approach. This is attributed to a combination of effects including a reduction in *p*-H_2_ enrichment, less efficient mixing, and longer transfer times during which the hyperpolarized signals will decay due to NMR relaxation. Nevertheless, the enhancement factors are significant (1200 fold for the example in Fig. 5B) and the flow-based approach provides the benefit of software control over parameters such as: *p*-H_2_ bubbling time, sample transfer time, and polarization transfer field. This control yields highly reproducible SABRE enhancement factors, a requirement for more advanced 2D experiments using SABRE and for quantitative applications. The reproducibility provided by the automated approach is demonstrated by the enhancement factors for 30 repeat SABRE measurements in Fig. 5C, where the sample was re-polarized using the automated system between each measurement. The relative standard deviation over the 30 repeat measurements is 5.0%, 5.3%, and 5.7% for the *ortho*, *meta* and *methyl*^1^H resonances, respectively. Despite this variation in the absolute level of SABRE enhancement, Fig. 5D shows that the distribution of the polarization within the analyte molecule is highly reproducible. When normalized to the enhancement of the largest peak, the *ortho*^1^H resonance, the relative enhancements of the *meta* and *methyl*^1^H resonances have a relative standard deviation of only 0.6% and 0.9%, respectively.

**Fig. 6:**
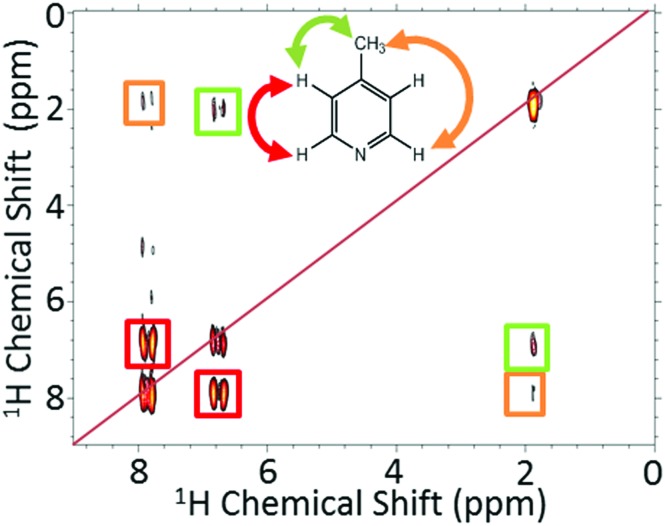
2D gradient selective COSY coupled with SABRE for 52 mM 4-methylpyridine and 5.2 mM catalyst in methanol-*d*_4_. The COSY measurements used 64 increments over a bandwidth of 500 Hz (11.5 ppm), with single scan transients and re-hyperpolarization between each point. The total experiment time was around 36.4 minutes.

Using high-field NMR detection, it has been shown previously that 2D SABRE hyperpolarized experiments such as ^1^H–^1^H COSY and ^13^C–^1^H HMQC^62^ and 2D DOSY^72^ are possible using the automated flow approach or using ultrafast single-shot methods with SABRE.^73^ Here we demonstrate that SABRE hyperpolarized 2D NMR can also be achieved using a benchtop NMR spectrometer for detection. Fig. 6 presents a SABRE hyperpolarized 2D gradient selective COSY spectrum of 52 mM of 4-methylpyridine acquired with 64 points in the indirect dimension, with re-hyperpolarization of the sample between each step (see ESI† for pulse sequence). The resulting COSY spectrum in Fig. 6 shows the expected peak patterns with the three resonances on the diagonal and as well as off-diagonal peaks indicative of the coupling between these resonances.

## Conclusions

Portable, low cost, benchtop NMR spectrometers already show great promise for many analytical applications but are limited by low sensitivity. In this work we have demonstrated the potential of the *p*-H_2_ based SABRE hyperpolarization technique to overcome this sensitivity limitation. Specifically, we have demonstrated a 17 000-fold increase in the signal strength for a benchtop (1 T) NMR spectrometer for ^1^H and a 45 500-fold increase for natural abundance ^13^C, which allows for low concentration analyte detection. These large signal enhancements are possible because, as demonstrated herein, the level of SABRE-generated hyperpolarization is independent of the detection field, with comparable results obtained at 400 MHz (9.4 T) and 43 MHz (1 T). The combination of SABRE with a benchtop NMR spectrometer has the added advantage of enabling the use of protonated solvents due to the relatively weak solvent signals at low field. This suggests SABRE hyperpolarized benchtop NMR may be implemented without the need for costly and time consuming sample preparation steps. One disadvantage of ^1^H detection at lower field is reduced chemical shift dispersion. However the chemical shift dispersions of nuclei such as ^13^C are larger, allowing much more diagnostic spectra to be obtained. We have demonstrated that natural abundance ^13^C{^1^H} spectra at 1 T are possible with good signal to noise in as little as 15 seconds using SABRE hyperpolarization. Comparable thermally polarized ^13^C NMR spectra at 1 T require either highly concentrated samples and long experiment times (as demonstrated here) or isotopic labelling.

The example analytes used herein to illustrate the potential of this method were optimized to provide signal enhancements by factors of more than 10^4^. We anticipate even with the more modest enhancements (on the order of 100–1000 fold) that could be routinely achieved for a broader range of analytes, the use of SABRE has the potential to open up exciting analytical applications by bridging the sensitivity gap between benchtop and high-field NMR spectroscopy. Furthermore, with the recent introduction of the SABRE-Relay method, the range of analytes amenable to hyperpolarization has been dramatically increased and now includes molecules with exchangeable protons such as amines, amides, alcohols, carboxylic acids, phosphates and carbonates.^38^ This approach has already been shown to work with ^1^H, ^13^C, ^15^N, ^19^F and ^31^P responses and a future truly versatile low field platform can now be envisaged.

By integrating an automated polarization system with the 1 T benchtop NMR spectrometer, we have shown how the continuous nature of the SABRE process can be exploited to repeatedly re-polarize the sample on a timescale of tens of seconds. The automated system allows for software control over SABRE parameters, namely: the bubbling time, transfer time, and polarization transfer field. This results in reproducible levels of total hyperpolarization as well as a highly consistent distribution of polarization within the target analyte. This reproducible SABRE hyperpolarization was exploited to carry-out a SABRE-enhanced 2D COSY NMR by transferring the sample back and forth to the polarizing unit for repolarization between each step of the experiment. The increased resolution which can be achieved from ^13^C and 2D experiments means that enhanced benchtop NMR now has the potential to be both highly specific and highly sensitive and therefore offers the ability to detect, identify, and quantify analytes at low concentration in complex mixtures (where many other components might be SABRE active in the ^1^H spectrum). Given the number of 2D sequences that have been shown to be amenable to this technique at high field (*e.g.* DOSY^72^ and HMBC^62^) and progress towards quantitative analysis with SABRE,^74^ we believe that this technology has the potential for rapid development into a versatile low cost technique for use in many analytical applications.

## Data Access Statement

All experimental NMR data reported in this work is available *via* Research Data York at ; http://dx.doi.org/10.15124/01dbd626-1e7a-473f-81bd-1ede6b3a935d.

## Conflicts of interest

There are no conflicts to declare.

## Supplementary Material

Supplementary information

## References

[cit1] Levitt M. H., Spin dynamics: basics of nuclear magnetic resonance, John Wiley & Sons, 2001.

[cit2] Colnago L. A., Andrade F. D., Souza A. A., Azeredo R. B. V., Lima A. A., Cerioni L. M., Osan T. M., Pusiol D. J. (2014). Chem. Eng. Technol..

[cit3] Dalitz F., Cudaj M., Maiwald M., Guthausen G. (2012). Prog. Nucl. Magn. Reson. Spectrosc..

[cit4] Blümich B. (2016). Trends Anal. Chem..

[cit5] Mitchell J., Gladden L. F., Chandrasekera T. C., Fordham E. J. (2014). Prog. Nucl. Magn. Reson. Spectrosc..

[cit6] Blümich B., Singh K. (2017). Angew. Chem., Int. Ed..

[cit7] van Duynhoven J., Voda A., Witek M., Van As H. (2010). Annu. Rep. NMR Spectrosc..

[cit8] Brown R. J. S., Chandler R., Jackson J. A., Kleinberg R. L., Miller M. N., Paltiel Z., Prammer M. G. (2001). Concepts Magn. Reson..

[cit9] Zalesskiy S. S., Danieli E., Blümich B., Ananikov V. P. (2014). Chem. Rev..

[cit10] Sans V., Porwol L., Dragone V., Cronin L. (2015). Chem. Sci..

[cit11] Singh K., Blümich B. (2017). Analyst.

[cit12] Friebel A., Froscher A., Munnemann K., von Harbou E., Hasse H. (2017). Fluid Phase Equilib..

[cit13] Halse M. E. (2016). Trends Anal. Chem..

[cit14] Lee J. H., Okuno Y., Cavagnero S. (2014). J. Magn. Reson..

[cit15] Nikolaou P., Goodson B. M., Chekmenev E. Y. (2015). Chem. – Eur. J..

[cit16] Slichter C. P. (2014). Rep. Prog. Phys..

[cit17] Ardenkjaer-Larsen J. H., Fridlund B., Gram A., Hansson G., Hansson L., Lerche M. H., Servin R., Thaning M., Golman K. (2003). Proc. Natl. Acad. Sci. U. S. A..

[cit18] Goodson B. M. (2002). J. Magn. Reson..

[cit19] Adams R. W., Aguilar J. A., Atkinson K. D., Cowley M. J., Elliott P. I. P., Duckett S. B., Green G. G. R., Khazal I. G., López-Serrano J., Williamson D. C. (2009). Science.

[cit20] Adams R. W., Duckett S. B., Green R. A., Williamson D. C., Green G. G. (2009). J. Chem. Phys..

[cit21] Atkinson K. D., Cowley M. J., Duckett S. B., Elliott P. I. P., Green G. G. R., Lopez-Serrano J., Khazal I. G., Whitwood A. C. (2009). Inorg. Chem..

[cit22] Truong M. L., Theis T., Coffey A. M., Shchepin R. V., Waddell K. W., Shi F., Goodson B. M., Warren W. S., Chekmenev E. Y. (2015). J. Phys. Chem. C.

[cit23] Duckett S. B., Wood N. J. (2008). Coord. Chem. Rev..

[cit24] Roy S. S., Rayner P. J., Norcott P., Green G. G. R., Duckett S. B. (2016). Phys. Chem. Chem. Phys..

[cit25] Coffey A. M., Shchepin R. V., Feng B. B., Colon R. D., Wilkens K., Waddell K. W., Chekmenev E. Y. (2017). J. Magn. Reson..

[cit26] Roth M., Kindervater P., Raich H. P., Bargon J., Spiess H. W., Munnemann K. (2010). Angew. Chem., Int. Ed..

[cit27] Natterer J., Bargon J. (1997). Prog. Nucl. Magn. Reson. Spectrosc..

[cit28] Green R. A., Adams R. W., Duckett S. B., Mewis R. E., Williamson D. C., Green G. G. (2012). Prog. Nucl. Magn. Reson. Spectrosc..

[cit29] Tom B. A., Bhasker S., Miyamoto Y., Momose T., McCall B. J. (2009). Rev. Sci. Instrum..

[cit30] Hovener J. B., Bar S., Leupold J., Jenne K., Leibfritz D., Hennig J., Duckett S. B., von Elverfeldt D. (2013). NMR Biomed..

[cit31] Bowers C. R., Weitekamp D. P. (1987). J. Am. Chem. Soc..

[cit32] Pravica M. G., Weitekamp D. P. (1988). Chem. Phys. Lett..

[cit33] Theis T., Truong M. L., Coffey A. M., Shchepin R. V., Waddell K. W., Shi F., Goodson B. M., Warren W. S., Chekmenev E. Y. (2015). J. Am. Chem. Soc..

[cit34] Ducker E. B., Kuhn L. T., Munnemann K., Griesinger C. (2012). J. Magn. Reson..

[cit35] Atkinson K. D., Cowley M. J., Elliott P. I. P., Duckett S. B., Green G. G. R., Lopez-Serrano J., Whitwood A. C. (2009). J. Am. Chem. Soc..

[cit36] Cowley M. J., Adams R. W., Atkinson K. D., Cockett M. C. R., Duckett S. B., Green G. G. R., Lohman J. A. B., Kerssebaum R., Kilgour D., Mewis R. E. (2011). J. Am. Chem. Soc..

[cit37] Rayner P. J., Burns M. J., Olaru A. M., Norcott P., Fekete M., Green G. G. R., Highton L. A. R., Mewis R. E., Duckett S. B. (2017). Proc. Natl. Acad. Sci. U. S. A..

[cit38] Iali W., Rayner P. J., Duckett S. B. (2018). Sci. Adv..

[cit39] Shchepin R. V., Goodson B. M., Theis T., Warren W. S., Chekmenev E. Y. (2017). ChemPhysChem.

[cit40] Burns M. J., Rayner P. J., Green G. G. R., Highton L. A. R., Mewis R. E., Duckett S. B. (2015). J. Phys. Chem. B.

[cit41] Zhivonitko V. V., Skovpin I. V., Koptyug I. V. (2015). Chem. Commun..

[cit42] Olaru A. M., Burt A., Rayner P. J., Hart S. J., Whitwood A. C., Green G. G. R., Duckett S. B. (2016). Chem. Commun..

[cit43] Barskiy D. A., Shchepin R. V., Tanner C. P. N., Colell J. F. P., Goodson B. M., Theis T., Warren W. S., Chekmenev E. Y. (2017). ChemPhysChem.

[cit44] Roy S. S., Norcott P., Rayner P. J., Green G. G. R., Duckett S. B. (2017). Chem. – Eur. J..

[cit45] Hovener J. B., Schwaderlapp N., Borowiak R., Lickert T., Duckett S. B., Mewis R. E., Adams R. W., Burns M. J., Highton L. A. R., Green G. G. R., Olaru A., Hennig J., von Elverfeldtt D. (2014). Anal. Chem..

[cit46] Roy S. S., Stevanato G., Rayner P. J., Duckett S. B. (2017). J. Magn. Reson..

[cit47] Pravdivtsev A. N., Yurkovskaya A. V., Zimmermann H., Vieth H.-M., Ivanov K. L. (2015). RSC Adv..

[cit48] Mewis R. E., Atkinson K. D., Cowley M. J., Duckett S. B., Green G. G., Green R. A., Highton L. A., Kilgour D., Lloyd L. S., Lohman J. A., Williamson D. C. (2014). Magn. Reson. Chem..

[cit49] Barskiy D. A., Kovtunov K. V., Koptyug I. V., He P., Groome K. A., Best Q. A., Shi F., Goodson B. M., Shchepin R. V., Truong M. L., Coffey A. M., Waddell K. W., Chekmenev E. Y. (2014). ChemPhysChem.

[cit50] Shchepin R. V., Barskiy D. A., Coffey A. M., Feldman M. A., Kovtunova L. M., Bukhtiyarov V. I., Kovtunov K. V., Goodson B. M., Koptyug I. V., Chekmenev E. Y. (2017). ChemistrySelect.

[cit51] Gloggler S., Emondts M., Colell J., Muller R., Blumich B., Appelt S. (2011). Analyst.

[cit52] Borowiak R., Schwaderlapp N., Huethe F., Lickert T., Fischer E., Bar S., Hennig J., von Elverfeldt D., Hovener J. B. (2013). Magn. Reson. Mater. Phys., Biol. Med..

[cit53] Turschmann P., Colell J., Theis T., Blümich B., Appelt S. (2014). Phys. Chem. Chem. Phys..

[cit54] Tayler M. C. D., Theis T., Sjolander T. F., Blanchard J. W., Kentner A., Pustelny S., Pines A., Budker D. (2017). Rev. Sci. Instrum..

[cit55] McDermott R., Trabesinger A. H., Muck M., Hahn E. L., Pines A., Clarke J. (2002). Science.

[cit56] Ledbetter M. P., Theis T., Blanchard J. W., Ring H., Ganssle P., Appelt S., Blumich B., Pines A., Budker D. (2011). Phys. Rev. Lett..

[cit57] Buckenmaier K., Rudolph M., Back C., Misztal T., Bommerich U., Fehling P., Koelle D., Kleiner R., Mayer H. A., Scheffler K., Bernarding J., Plaumann M. (2017). Sci. Rep..

[cit58] Theis T., Ledbetter M. P., Kervern G., Blanchard J. W., Ganssle P. J., Butler M. C., Shin H. D., Budker D., Pines A. (2012). J. Am. Chem. Soc..

[cit59] Shchepin R. V., Coffey A. M., Waddell K. W., Chekmenev E. Y. (2014). Anal. Chem..

[cit60] Spannring P., Reile I., Emondts M., Schleker P. P., Hermkens N. K., van der Zwaluw N. G., van Weerdenburg B. J., Tinnemans P., Tessari M., Blümich B., Rutjes F. P., Feiters M. C. (2016). Chem. – Eur. J..

[cit61] Richardson P. M., Jackson S., Parrott A. J., Nordon A., Duckett S. B., Halse M. E. (2018). Magn. Reson. Chem..

[cit62] Lloyd L. S., Adams R. W., Bernstein M., Coombes S., Duckett S. B., Green G. G., Lewis R. J., Mewis R. E., Sleigh C. J. (2012). J. Am. Chem. Soc..

[cit63] Cowley M. J., Adams R. W., Atkinson K. D., Cockett M. C., Duckett S. B., Green G. G., Lohman J. A., Kerssebaum R., Kilgour D., Mewis R. E. (2011). J. Am. Chem. Soc..

[cit64] Appleby K. M., Mewis R. E., Olaru A. M., Green G. G. R., Fairlamb I. J. S., Duckett S. B. (2015). Chem. Sci..

[cit65] Kuhn L. T., Hyperpolarization Methods in NMR Spectroscopy, Springer-Verlag, Berlin Heidelberg, 2013.

[cit66] Mewis R. E. (2015). Magn. Reson. Chem..

[cit67] Shi F., Coffey A. M., Waddell K. W., Chekmenev E. Y., Goodson B. M. (2015). J. Phys. Chem. C.

[cit68] Bloembergen N., Pound R. V. (1954). Phys. Rev..

[cit69] Gueron M., Plateau P., Decorps M. (1991). Prog. Nucl. Magn. Reson. Spectrosc..

[cit70] Simpson A. J., Brown S. A. (2005). J. Magn. Reson..

[cit71] Zheng G., Price W. S. (2010). Prog. Nucl. Magn. Reson. Spectrosc..

[cit72] Reile I., Aspers R., Tyburn J. M., Kempf J. G., Feiters M. C., Rutjes F., Tessari M. (2017). Angew. Chem., Int. Ed..

[cit73] Daniele V., Legrand F. X., Berthault P., Dumez J. N., Huber G. (2015). ChemPhysChem.

[cit74] Eshuis N., Hermkens N., van Weerdenburg B. J., Feiters M. C., Rutjes F. P., Wijmenga S. S., Tessari M. (2014). J. Am. Chem. Soc..

