# Supplementary material for: SABRE hyperpolarization enables high-sensitivity 1H and 13C benchtop NMR spectroscopy
Source: Analyst. 2018 Jun 19;143(14):3442–50. doi: 10.1039/c8an00596f (PMC6040279; doi:10.1039/c8an00596f)
Supplement: Supplementary file 1 [file AN-143-C8AN00596F-s001.pdf]

This journal is © The Royal Society of Chemistry 2018

Supporting Information

SABRE hyperpolarization enables high-sensitivity ^{1}H and ^{13}C benchtop NMR spectroscopy

Peter M. Richardson†, Andrew J. Parrott ‡, Olga Semenova†, Alison Nordon‡, Simon B. Duckett†* and Meghan E. Halse†*

† Centre for Hyperpolarisation in Magnetic Resonance, Department of Chemistry, University of York, UK

‡ WestCHEM, Department of Pure and Applied Chemistry and CPACT, University of Strathclyde, Glasgow, UK.

Enhancement Factor Calculation 2

Polarization Calculation 2

Reference Thermally-polarized ^{1}H NMR Integrals 2

^{13}C Enhancement Values 4

^{13}C Chemical Shift Dependencies 7

2D Gradient Selective COSY Pulse Sequence 9

Automated Flow System Pulse Sequences 10

Enhancement Factor Calculation

The enhancement factors were determined by taking a ratio of the integrals of the relevant peak in the hyperpolarized NMR spectrum and the thermally polarized NMR spectrum according to equation S1.

$$\epsilon = \frac{\text{integral of hyperpolarized NMR spectrum}}{\text{integral of thermal NMR spectrum}} \qquad \text{(S1)}$$

Polarization Calculation

In order to more easily compare the enhancements at 9.4 T and 1 T the enhancement factor, ϵ was converted to a polarization level, P, using Eq. S2, where, γ is the gyromagnetic ratio, B_0 is the detection field, T is the temperature, ħ is the reduced Planck's constant, and k_B is Boltzmann's constant.

$$P = \epsilon \frac{\gamma B_0 \hbar}{2 k_B T} \qquad \text{(S2)}$$

Reference Thermally-polarized ^{1}H NMR Integrals

In order to minimize error in the reference NMR signal measurements, especially at low concentrations of analyte, a least squares linear fit was carried out to determine the correlation between the integral of a thermally-polarized ^{1}H NMR spectrum and the analyte concentration. The correlation was carried out for the *ortho* resonance of the two analytes used in this study (pyridine and 4-methylpyridine) as this resonance does not overlap with any other peaks from the active catalyst or the solvent. These reference measurements were carried out at 1 T and 9.4 T for pyridine (Figure S1) and at 1 T for 4-methylpyridine (Figure S2). As expected, the correlation is the same to within expected experimental error for both analytes at 1 T. The calibration experiments were carried out in deuterated methanol in all cases. The gradient, G, and intercept from the calibration curve, I, was used to determine the denominator in equation S1 according to equation S3, where *G* is the gradient of the calibration curve in mM^{-1}, [S] is the concentration of the substrate in the SABRE experiment in mM and *N* is the number of ^{1}H's associated with the given resonance in the SABRE spectrum (i.e. *N* = 3 for the *methyl* resonance and N = 2 for *meta* resonance in 4-methylpyridine.)

$$reference\ integral = (G[S] + I)\left(\frac{N}{2}\right) \qquad (S3)$$

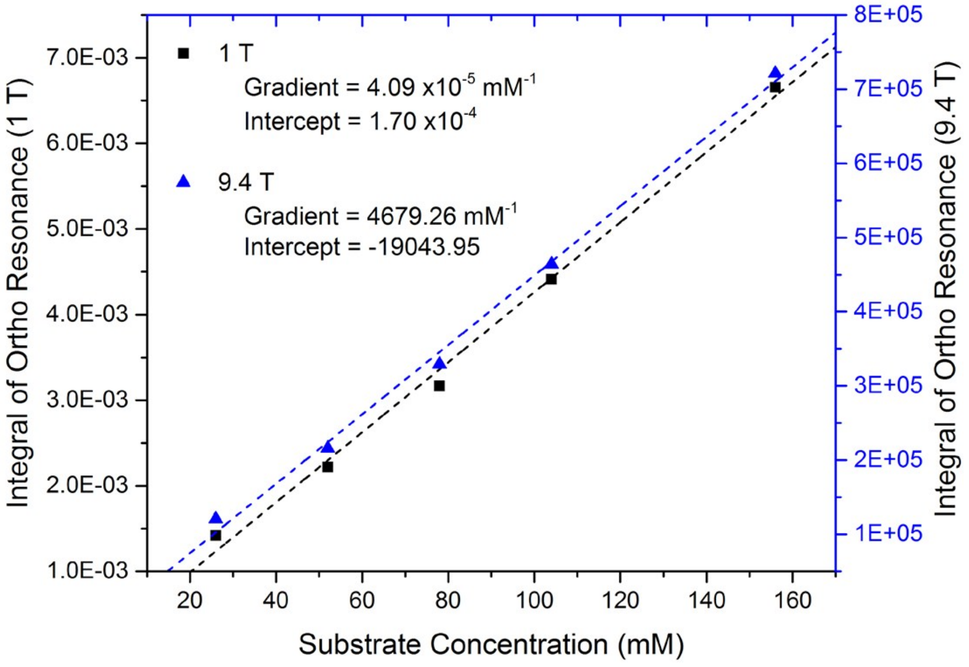

Figure S1. Integrals of thermally-polarized ^{1}H NMR spectra measured at 400 MHz (9.4 T, right, blue triangles) and 43 MHz (1 T, left, black squares) for the ortho ^{1}H resonance of pyridine in methanol-d_4 with 5.2 mM of [IrCl(COD)(IMes)]. The line-of-best-fit was used to determine the thermal ^{1}H NMR signal for the calculation of hyperpolarization enhancement factors.

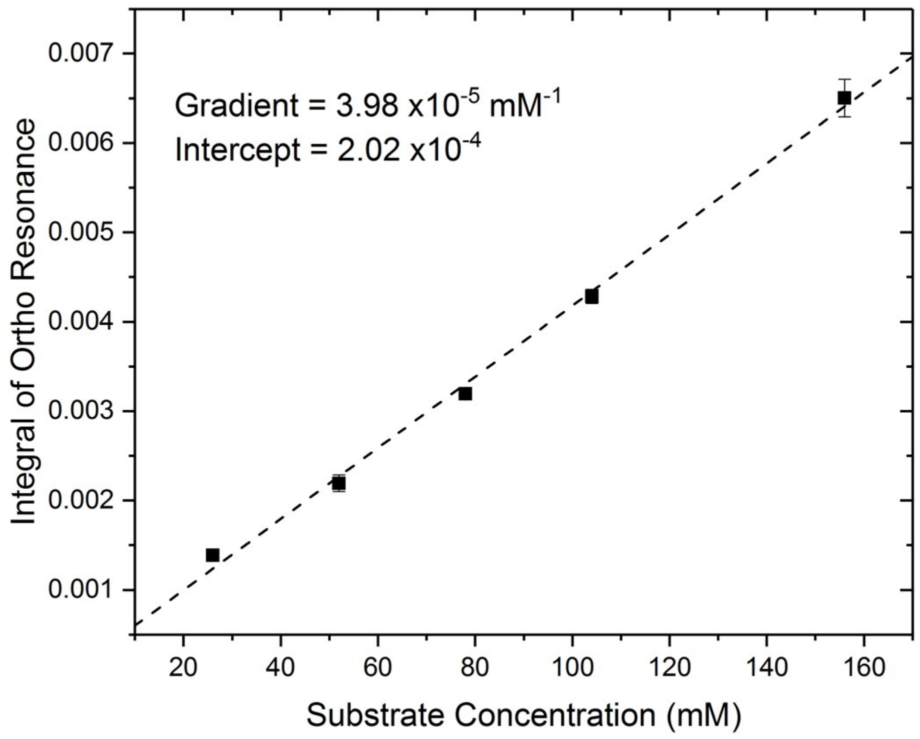

Figure S2. Thermal integrals measured on a 43 MHz (1 T) spectrometer for the ortho resonance of 4-methylpyridine in methanol-d_4 with 5.2 mM of [IrCl(COD)(IMes)]. The line-of-best-fit was used to determine the thermal NMR signal per unit concentration in the calculations of the hyperpolarisation enhancement factors.

^{13}C Enhancement Values

When the ^{13}C NMR spectrum of 4-methylpyridine is fully coupled there are nine peaks that can be analyzed. The peaks at 156.5 ppm and 140.2 ppm for the *ortho* resonance (C_a) are separated by the C-H splitting of 178 Hz, which corresponds to 16.3 ppm at this detection field. Analogously, the peaks at 132.5 ppm and 117.6 ppm correspond to the C-H partners of the *meta* ^{13}C resonance (C_b) and yield a $^1J_{CH}$ value of 163 Hz. ^{13}C nuclei in the *para* position (C_c) appear as a multiplet at 148.9 ppm and the remaining peaks at 37.1 ppm, 25.5 ppm, 13.9 ppm and 2.2 ppm arise from the methyl resonance whose $^1J_{CH}$ value is 127 Hz. The fine structure evident in these resonances reflects additional two and three-bond ^{1}H-^{13}C couplings.

The NMR integrals for each anti-phase peak in the fully-coupled SABRE-enhanced ^{13}C NMR spectra were calculated by separately integrating the two halves of the anti-phase peak and taking the difference between them. The individual integrals for each part of the multiplet corresponding to a single ^{13}C site was then added together (i.e. the total integral for the C_a resonance was calculated as the sum of the anti-phase integrals of the peak at 156.5 ppm and at 140.2 ppm). The signal enhancement factor was then estimated using equation S4 where n is the number of transients for the reference spectrum, $[S]_{ref}$ is the concentration of the analyte in the reference sample, and $[S]_{SABRE}$ is the concentration of the analyte in the hyperpolarized spectrum.

$$\epsilon = \frac{\text{integral of hyperpolarized NMR spectrum}}{\text{integral of the reference NMR spectrum}} \frac{[S]_{ref}}{[S]_{SABRE}} n \qquad (S4)$$

The ^{13}C enhancement factors and corresponding polarization levels (calculated using equation S2) for the fully-coupled ^{13}C NMR spectra in Figure 4A in the text, where the reference spectrum was acquired with n = 4096 transients on a neat sample of natural abundance 4-methylpyridine with a concentration of 10.276 M in methanol-d_4, are summarized in Figure S3.

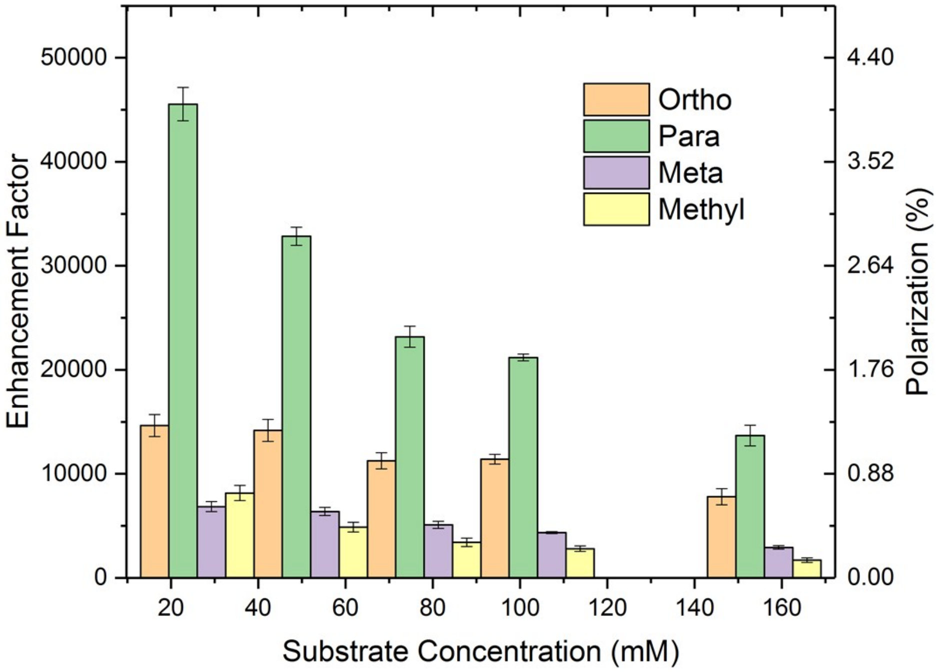

Figure S3. ¹³C SABRE NMR enhancements for varying concentration of 4-methylpyridine with 5.2 mM [IrCl(COD)(IMes)] catalyst in methanol-d_4. Error bars depict the standard deviation over 4 repeated SABRE hyperpolarization experiments.

The ¹³C enhancement factors and corresponding polarization levels in protio methanol are summarized in Figure S4. Again, all resonances receive hyperpolarization; however, the polarization levels are lower than the corresponding samples in methanol-d_4. This was attributed to the reduced hyperpolarization lifetimes in a protonated versus a deuterated solvent.

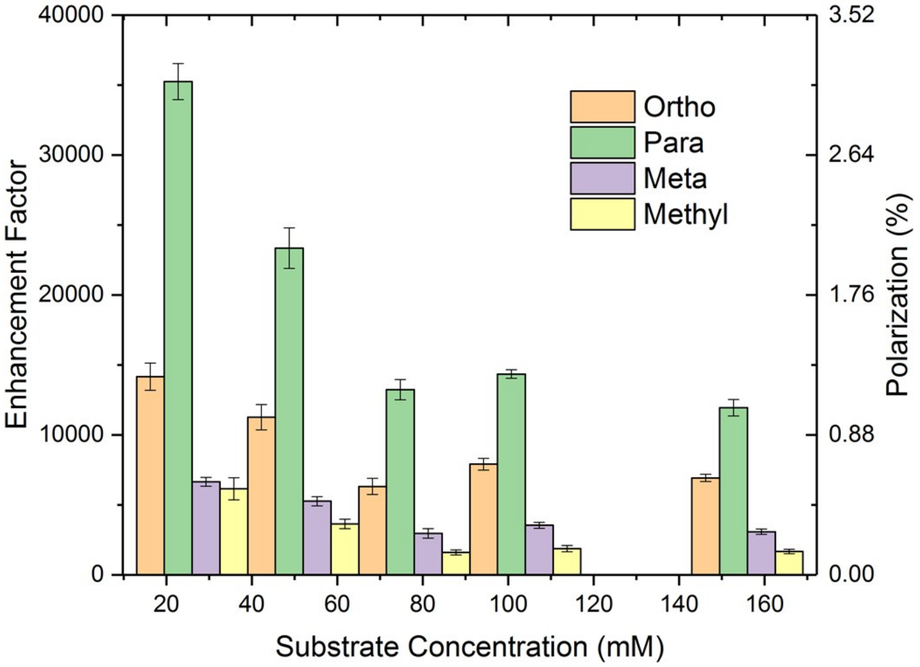

Figure S4. ¹³C SABRE NMR enhancements for varying concentration of 4-methylpyridine with 5.2 mM [IrCl(COD)(IMes)] catalyst in protonated methanol. Error bars depict the standard deviation over 4 repeated SABRE hyperpolarization experiments.

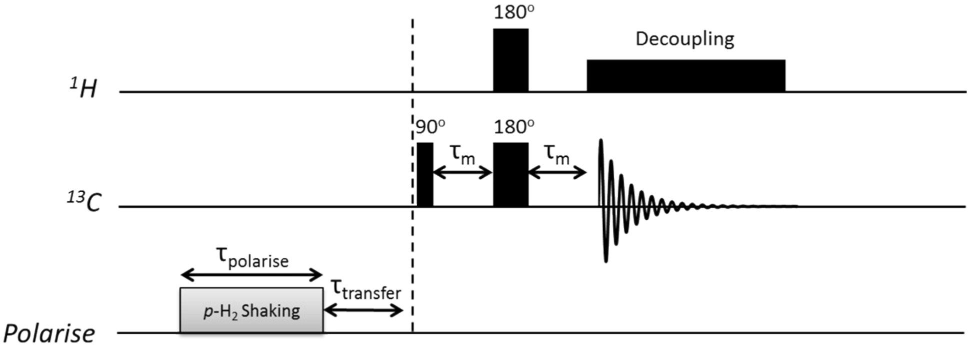

Figure S5. In order to decouple the anti-phase SABRE experiment the signal must first be refocussed otherwise the signal will be cancelled. The pulse sequence shown was used in order to obtain ¹³C{¹H} spectra. The SABRE hyperpolarisation is first achieved by shaking the sample in a small magnetic field for a given time ($\tau_{polarise}$) under a pressure of para-hydrogen gas. The sample is then transferred into the NMR spectrometer in time $\tau_{transfer}$. Once in the spectrometer, a 90° pulse on the ¹³C channel is applied followed by a mixing time which is defined here as $\tau_m = 1/4J_{CH}$, where $^2J_{CH} = 10.5$ Hz. Then 180°

pulses are applied on the ^{1}H and ^{13}C channels to complete the refocussing, followed by a final mixing time delay before acquisition.

The SABRE enhancement factors and polarization levels for the decoupled ^{13}C NMR spectra were calculated using equations S2 and S4, where the reference ^{13}C NMR spectrum (top in Figure 4B in the text) was acquired with $n = 256$ transients on a neat sample of natural abundance 4-methylpyridine with a concentration of 10.276 M. The resultant SABRE enhancement factors and polarization levels are summarized in table S1.

Table S1. Summary of natural abundance $^{13}C\{^{1}H\}$ SABRE NMR hyperpolarization values for 156 mM 4-methylpyridine with 5.2 mM of catalyst in methanol-d4 obtained with NMR signal detection in a benchtop (1T) NMR spectrometer.

	**Chemical shift (ppm)**	**Enhancement Factor**	**Polarization (%)**
**Ortho (C_a)**	148.4	10,200	0.90
**Para (C_b)**	148.9	17,000	1.49
**Meta (C_c)**	125.1	4,600	0.41
**Methyl (C_d)**	19.7	7,600	0.67

^{13}C Chemical Shift Dependencies

When comparing the thermal NMR spectrum and the corresponding SABRE enhanced signal, there was a change in chemical shift, most notably for the carbon in the *para* position of the substrate (C_c). There were two factors that differed between the SABRE sample and the sample used in taking the thermal reference spectrum. Firstly, the temperature of the Magritek benchtop system is held at 301 K (28°C), therefore the reference NMR spectra were taken at this temperature, whereas the SABRE sample was polarized outside the magnet in the lab, which is maintained at 293 K (20°C), resulting in a small temperature difference. Secondly, due to the low natural abundance of ^{13}C coupled with the reduced signal observed on a 1 T NMR spectrometer a neat sample of 4-methylpyridine was used to take the reference spectrum, whereas the SABRE sample contained 156 mM of the substrate in methanol.

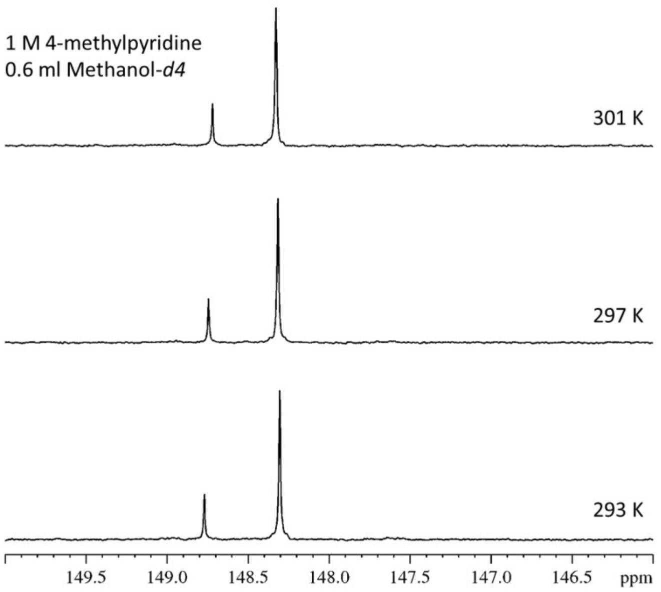

Figure S6. $^{13}C\{^1H\}$ NMR spectra of 1 M 4-methylpyridine in 0.6 mL of deuterated methanol at 293 K, 297 K and 301 K. All were measured using a Bruker 500 MHz NMR spectrometer using a standard $^{13}C\{^1H\}$ pulse sequence with a 30° excitation pulse. The spectral range has been selected to just show the ortho and para position carbons on the substrate ring.

These two factors were investigated using a Bruker 500 MHz spectrometer. Figure S4 shows the *ortho* and *para* region of $^{13}C\{^1H\}$ NMR spectra of 1 M 4-methylpyridine in methanol-d_4 (CD_3OD) at three different temperatures varying from the temperature of the lab up to the temperature of the Magritek benchtop NMR spectrometer. The observed chemical shift change on increasing the temperature from 293 K to 301 K was +0.02 ppm, -0.05 ppm, -0.03 ppm and -0.04 ppm for the carbon nuclei in the *ortho*, *para*, *meta* and *methyl* positions of the substrate, respectively. Therefore the possible temperature difference between the SABRE and reference experiments was not the cause of the observed peak shifts.

The effect of the concentration of substrate was also investigated using three different samples: neat 4-methylpyridine (10.28 M), 1 M 4-methylpyridine in deuterated methanol and 156 mM 4-methylpyridine in deuterated methanol; the latter is the same concentration as that used in the SABRE experiments. Figure S7 shows the *para* and *ortho* resonances in the $^{13}C\{^1H\}$ NMR spectra at

each concentration of 4-methylpyridine as well as a fully-coupled ^{13}C NMR spectrum for the neat 4-methylpyridine sample.

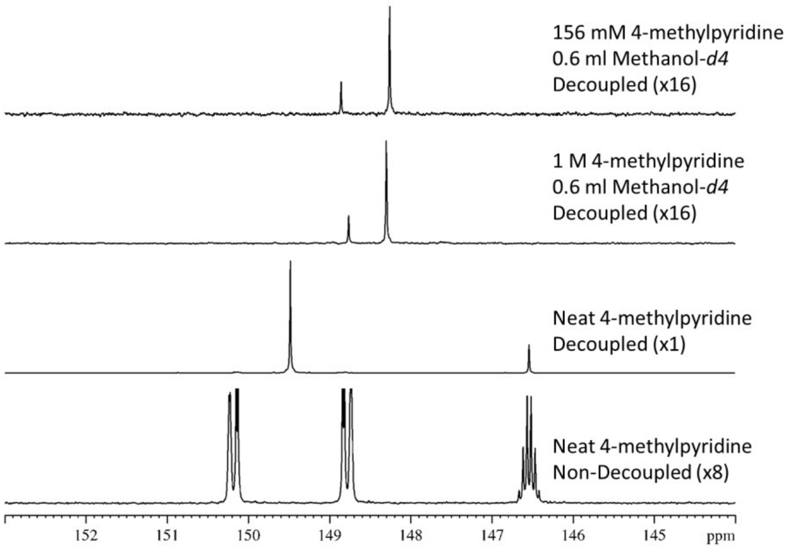

Figure S7 – $^{13}C\{^1H\}$ NMR spectra with varying concentrations of 4-methylpyridine in methanol-d_4 at 293 K. All were measured using a Bruker 500 MHz NMR spectrometer, using a standard $^{13}C\{^1H\}$ pulse sequence with a 30° excitation pulse for the top three spectra and a fully coupled equivalent for the bottom spectrum, also using a 30° excitation pulse. The spectral range has been selected to show the ortho and para position carbons on the substrate ring.

There is a significant change in chemical shift on changing the concentration of 4-methylpyridine in methanol-d_4. The chemical shift differences between neat and 156 mM 4-methlypyridine (i.e. the concentration used in the ^{13}C SABRE experiments) are 1.23 ppm, 2.37 ppm, 0.51 ppm and 0.49 ppm for the *ortho*, *para*, *meta* and *methyl* positions, respectively. The chemical shifts obtained for 4-methlypyridine in the SABRE experiment at 1 T were 148.26 ppm, 148.86 ppm, 124.95 ppm and 19.61 ppm for the *ortho*, *para*, *meta* and *methyl*, respectively.

2D Gradient Selective COSY Pulse Sequence

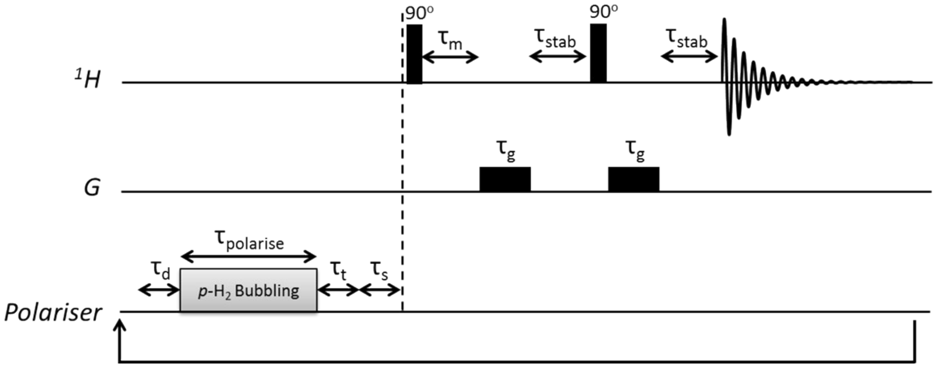

Figure S8. 2D gradient selective COSY sequence coupled with SABRE hyperpolarisation. At the beginning of each point a delay, τ_d, of 10 s was used in order to allow para-hydrogen levels to replenish. Then the polarisation step is carried out with 4 bar of para-hydrogen being bubbled through the solution with a magnetic field of around 65 G applied to the system for a time $\tau_{polarise}$ which was 15 seconds. Then the sample was transferred to the spectrometer in time τ_t then allowed for a settling time τ_s, where the transfer time was typically around 4 seconds and the settling time was set to 0.1 seconds. Then a standard gradient selective COSY sequence was applied to the system, followed by acquisition. The solution is then returned to the mixing chamber to be repolarized for the next mixing time in the experiment. The COSY shown in figure 6d was performed with 64 points in the indirectly detected dimension, each with a single transient, with re-hyperpolarisation between each point. Total experiment time was 36.4 minutes

Automated Flow System Pulse Sequences

At the heart of the automated flow system is a control unit developed as a result of a collaboration between the University of York and Bruker. In order to integrate the SABRE repolarisation step into each hyperpolarisation experiment on the Magritek Spinsolve spectrometer, a macro was written within the Spinsolve Expert software environment to pass commands from the controlling PC to the SABRE polarisation control unit. Communication was achieved using a RS232 serial interface and was mediated by the controlling PC, external to the execution of each step of the NMR sequence by the DSP of the Spinsolve spectrometer.

The integration of the SABRE polarisation step into each NMR pulse sequence is achieved as follows. At the beginning of each pulse sequence a set of user-define parameters (valve pressure and timings) are sent to the control unit via the serial interface. These parameters are optimized by the user to ensure that the sample is efficiently and reliably transferred between the reaction chamber and the flow cell within the NMR spectrometer. Within the main pulse sequence a polarisation step is included prior to the execution of each increment of the NMR experiment. Specifically, a single command is sent to initiate bubbling of p-H_2 through the sample solution within the reaction chamber. After the desired polarisation transfer time has elapsed, where the time delay is implemented within the Spinsolve macro environment, a command is sent to the control unit to stop the flow of p-H_2. This command triggers a fixed subroutine within the control unit that releases the p-H_2 pressure resulting in out-gassing of H_2 from the solution. This step takes a fixed period of approximately 3 s. Once the status of the control unit (queried from within the Spinsolve macro environment using the serial interface) indicates that the out-gassing is complete, the transfer of the sample from the reaction chamber to the spectrometer under a flow of N_2 is initiated by sending a single command to the control unit from within the Spinsolve macro environment. After the status of the control unit confirms that this operation is complete, and following a user-defined settling delay implemented within the Spinsolve macro, the execution of the NMR pulse sequence by the DSP within the spectrometer is initiated. Finally, after data acquisition is complete, a command is sent to the control unit to initiate the return of the sample to the reaction chamber under a flow of N_2. This process is repeated for each step of the NMR experiment, with an optional user-defined delay prior to the initialisation of p-H_2 bubbling to allow for the pressure of the p-H_2 to stabilize.

The precision of the timing of the different polarisation time elements (e.g. polarisation transfer time and sample transfer times) is limited by the speed of the RS232 serial interface. The exact timings for each step of the experiment are stored in a test file within the experimental data directory. The variability in the timings was typically found to be on the order of 10 ms, which is not significant relative to polarisation transfer and sample transfer times that are on the order of a few seconds. A more integrated implementation of the SABRE step, where the timings for the polarisation step are controlled by the 200 ns clock of the spectrometer DSP could be beneficial in order to improve the reproducibility of these pulse sequence elements.
